# Sagittal femoral morphology is not associated with coronal alignment phenotypes: A radiographic analysis from the Brescia Knee Research Group

**DOI:** 10.1002/jeo2.70869

**Published:** 2026-07-28

**Authors:** Luca Andriollo, Roberta Lazzaro, Michele Alborghetti, Lorenzo Elia, Giulio Calandra, Marina Marescalchi, Antonio Rapisarda, Roberto Perulli, Francesco Benazzo, Rudy Sangaletti

**Affiliations:** ^1^ Brescia Knee Research Group Fondazione Poliambulanza Istituto Ospedaliero Brescia Italy; ^2^ Ortopedia e Traumatologia Fondazione Poliambulanza Istituto Ospedaliero Brescia Italy; ^3^ Artificial Intelligence Center Alma Mater Europaea University Vienna Austria; ^4^ General Surgery, Department of Health Sciences University of Milan Milan Italy; ^5^ Biomedical Sciences Area IUSS University School for Advanced Studies Pavia Italy; ^6^ Department of Management, Information and Production Engineering University of Bergamo Dalmine Bergamo Italy; ^7^ Radiologia muscolo‐scheletrica Fondazione Poliambulanza Istituto Ospedaliero Brescia Italy

**Keywords:** coronal alignment phenotypes, CPAK, femoral offset, functional knee phenotypes, sagittal femoral morphology

## Abstract

**Purpose:**

To evaluate the association between sagittal femoral morphology and coronal alignment phenotypes. It was hypothesised that sagittal femoral morphology is not associated with coronal phenotype classifications.

**Methods:**

This retrospective study analysed a prospectively maintained institutional database including patients who underwent primary total knee arthroplasty (TKA) between January 2021 and November 2022. Preoperative radiographic assessment included standardised lateral and full‐length weight‐bearing radiographs. Coronal alignment parameters included mechanical hip–knee–ankle angle (mHKA), arithmetic hip–knee–ankle angle (aHKA), lateral distal femoral angle (LDFA), medial proximal tibial angle (MPTA) and joint line obliquity (JLO). Sagittal femoral morphology was assessed using anterior femoral offset (AFO), posterior femoral offset (PFO) and anteroposterior femoral size (AP size). Patients were classified according to the coronal plane alignment of the knee (CPAK) classification and functional knee phenotypes (FKPs).

**Results:**

A total of 212 patients were included. No significant associations were observed between sagittal femoral morphology and coronal phenotypes. AFO, PFO and AP size did not differ across CPAK types (*p* = 0.779, *p* = 0.127 and *p* = 0.124, respectively) or FKP groups (*p* = 0.622, *p* = 0.409 and *p* = 0.196, respectively). No significant differences were found when stratifying patients according to aHKA or JLO categories. Interaction analyses showed no significant effects between coronal phenotypes and sagittal morphology (all *p* > 0.05), with low explanatory power (*R* ^2^ ≤ 0.14).

**Conclusions:**

Sagittal femoral morphology is not associated with coronal alignment phenotypes, indicating that sagittal and coronal knee characteristics represent independent anatomical domains. Coronal phenotype classifications alone may therefore be insufficient to characterise patient‐specific femoral morphology during preoperative TKA planning.

**Level of Evidence:**

Level III.

**Abbreviations**
AFO: anterior femoral offset
aHKA: arithmetic hip–knee–ankle angle
AP size: anteroposterior femoral size
CI: confidence interval
CPAK: coronal plane alignment of the knee
FKP: functional knee phenotype
IRB: institutional review board
JLO: joint line obliquity
LDFA: lateral distal femoral angle
mHKA: mechanical hip–knee–ankle angle
MPTA: medial proximal tibial angle
OR: odds ratio
PFO: posterior femoral offset
PTS: posterior tibial slope
R^2^
: coefficient of determination
TKA: total knee arthroplasty

## INTRODUCTION

Total knee arthroplasty (TKA) planning increasingly incorporates patient‐specific anatomical characteristics rather than relying solely on uniform alignment targets [6, 8, 9]. In this context, substantial variability in native knee anatomy has been described, particularly in relation to coronal alignment patterns and joint‐line orientation [8, 9, 10, 13].

Coronal alignment variability has been extensively described using classifications such as the coronal plane alignment of the knee (CPAK) and functional knee phenotypes (FKPs), which incorporate constitutional alignment and joint‐line orientation [21, 26]. However, these systems are inherently limited to the coronal plane and do not account for the three‐dimensional morphology of the knee [3, 5, 14, 28].

Sagittal femoral morphology, including anterior femoral offset (AFO), posterior femoral offset (PFO) and anteroposterior femoral size (AP size), has been shown to influence knee kinematics, flexion gap behaviour, patellofemoral mechanics and post‐operative function after TKA [18, 23, 31]. However, its relationship with coronal phenotype classification remains unclear. Previous evidence suggests that coronal and sagittal alignment may represent largely independent anatomical domains [2, 4, 14, 15]. While previous studies have explored interactions between sagittal and coronal alignment parameters, little is known regarding whether contemporary coronal phenotype classifications such as CPAK and FKP are associated with sagittal femoral morphology.

The aim of this study was to evaluate the association between sagittal femoral morphology and coronal alignment phenotypes. It was hypothesised that sagittal femoral morphology would not be significantly associated with CPAK or FKP classifications.

## METHODS

This retrospective study was conducted using a prospectively maintained institutional database.

For this radiographic study, patients who underwent primary TKA between January 2021 and November 2022 were included. Patients were excluded if preoperative radiographic data were incomplete.

Baseline demographic data, including age and sex, were collected. Preoperative radiographic assessment included standardised anteroposterior, lateral, Rosenberg, skyline and full‐length weight‐bearing radiographs as part of the routine institutional protocol.

Measurements were recorded to the nearest 0.1 mm and 0.1°. The following coronal and sagittal parameters were measured: mechanical hip–knee–ankle angle (mHKA), lateral distal femoral angle (LDFA), medial proximal tibial angle (MPTA) and posterior tibial slope (PTS). Sagittal femoral morphology was assessed on standardised lateral radiographs using simplified two‐dimensional global sagittal morphology parameters, including AP size, AFO and PFO. AP size was defined as the anteroposterior distance between the most anterior aspect of the distal femur and the most posterior aspect of the posterior femoral condyles. AFO was defined as the perpendicular distance from the anterior femoral cortical reference line to the most anterior point of the distal anterior femur/trochlear region on standardised lateral radiographs. PFO was defined as the perpendicular distance from the posterior femoral cortical reference line to the most posterior aspect of the posterior femoral condyles on standardised lateral radiographs.

All measurements were performed using calibrated Picture Archiving and Communication System‐integrated digital measurement tools, based on the radiographic scaling embedded within the imaging system. All radiographic measurements were independently performed by two trained observers, with values recorded to the nearest 0.5°. Intra‐ and inter‐observer reliability were evaluated on a random sample of 50 patients, demonstrating excellent agreement, with intraclass correlation coefficient values of 0.93 (95% CI, 0.89–0.96) and 0.90 (95% CI, 0.85–0.94), respectively.

Patients were classified according to the CPAK classification and femoral FKP [21, 26].

The association between sagittal femoral morphology (AFO, PFO and AP size) and alignment phenotypes (CPAK classification and FKP) was evaluated.

Interaction analyses were performed to investigate whether these associations varied across different alignment phenotype subgroups.

### Ethical approval

This study was conducted in accordance with the Declaration of Helsinki and approved by the local Institutional Review Board (IRB Approval No. NK5022). Written informed consent was obtained from all participants.

### Statistical analysis

All statistical analyses were performed using MATLAB R2024b (MathWorks). Normality of continuous variables was assessed using the Lilliefors test. Data are presented as mean ± standard deviation or median with interquartile range, as appropriate. Categorical variables are reported as counts and percentages.

Between‐group comparisons were performed using the Kruskal–Wallis test. Associations were evaluated using linear regression models, with results reported as coefficients of determination (*R* ^2^). Interaction effects were assessed by including interaction terms within regression models. Associations with categorical variables were analysed using logistic regression models, with results expressed as odds ratios and 95% confidence intervals (CIs). A two‐sided *p* value < 0.05 was considered statistically significant.

As this was a retrospective analysis of a prospectively maintained database, no formal a priori sample size calculation was performed. In accordance with STROBE recommendations, all eligible patients during the study period were included. A sensitivity power analysis showed that, with 212 patients, *α* = 0.05 and 80% power, the study was able to detect a minimum correlation of approximately *r* = 0.19, corresponding to *R* ^2^ = 0.036. For an equivalent omnibus between‐group comparison, the detectable effect size was approximately Cohen's *f* = 0.27 for CPAK groups and *f* = 0.24 for FKP groups.

## RESULTS

At final follow‐up, a total of 212 patients were assessed. The baseline demographic and radiographic characteristics of the study population are summarised in Table 1.

**Table 1:** Baseline demographic data and preoperative radiographic measurements of the study population.

Variable	Value
Demographic data	
Number of patients (*n*)	212
Age at surgery (years)	71.0 [61.0–76.0]
Sex (male/female)	95 (44.8%)/117 (55.2%)
Side (right/left)	108 (50.9%)/104 (49.1%)
Coronal alignment parameters	
Mechanical hip–knee–ankle angle (°)	175.05° [170.70–181.35°]
Arithmetic hip–knee–ankle angle (°)	−1.35° [−4.80° to 3.55°]
Lateral distal femoral angle (°)	88.67 ± 3.46°
Medial proximal tibial angle (°)	87.85 ± 3.73°
Joint line obliquity (°)	176.30° [173.65–179.30°]
Sagittal alignment parameter	
Posterior tibial slope (°)	6.08 ± 4.08°
Sagittal femoral morphology	
Anteroposterior femoral size (mm)	71.94 ± 6.96
Anterior femoral offset (mm)	7.30 [5.60–8.85]
Posterior femoral offset (mm)	30.15 ± 5.02

The distribution of CPAK types in the study cohort was as follows: Type I (*n* = 61, 28.8%), Type II (*n* = 24, 11.3%), Type III (*n* = 37, 17.5%), Type IV (*n* = 30, 14.2%), Type V (*n* = 23, 10.8%), Type VI (*n* = 22, 10.4%), Type VII (*n* = 6, 2.8%), Type VIII (*n* = 2, 0.9%) and Type IX (*n* = 7, 3.3%).

The distribution of femoral FKP was as follows: VAL_FMA_6° (*n* = 1, 0.5%), VAL_FMA_3° (*n* = 9, 4.2%), NEU_FMA_0° (*n* = 34, 16.0%), VAR_FMA_3° (*n* = 70, 33.0%) and VAR_FMA_6° (*n* = 98, 46.2%).

### Association between sagittal femoral morphology and coronal phenotypes

No significant associations were observed between sagittal femoral morphology parameters and coronal phenotypes.

Sagittal femoral morphology parameters, including AFO, PFO and AP size, were compared across different coronal phenotype classifications. When stratified according to the CPAK classification, no significant differences were observed in AFO (*p* = 0.779), PFO (*p* = 0.127) or AP size (*p* = 0.124) among the different CPAK types.

Similarly, no statistically significant differences in sagittal femoral morphology were found across femoral FKP, with AFO (*p* = 0.622), PFO (*p* = 0.409) and AP size (*p* = 0.196) showing no variation between groups.

When patients were categorised according to aHKA, into varus (<−2°), neutral (−2° to +2°) and valgus (>+2°), no significant differences were observed in AFO (*p* = 0.910), PFO (*p* = 0.881) or AP size (*p* = 0.145) across the three groups.

Likewise, when joint line obliquity (JLO) was classified into apex distal (<177°), neutral (177–183°) and apex proximal (>183°), no significant differences in sagittal femoral morphology were found, with AFO (*p* = 0.628), PFO (*p* = 0.392) and AP size (*p* = 0.330) remaining comparable among groups.

**Figure 1:**
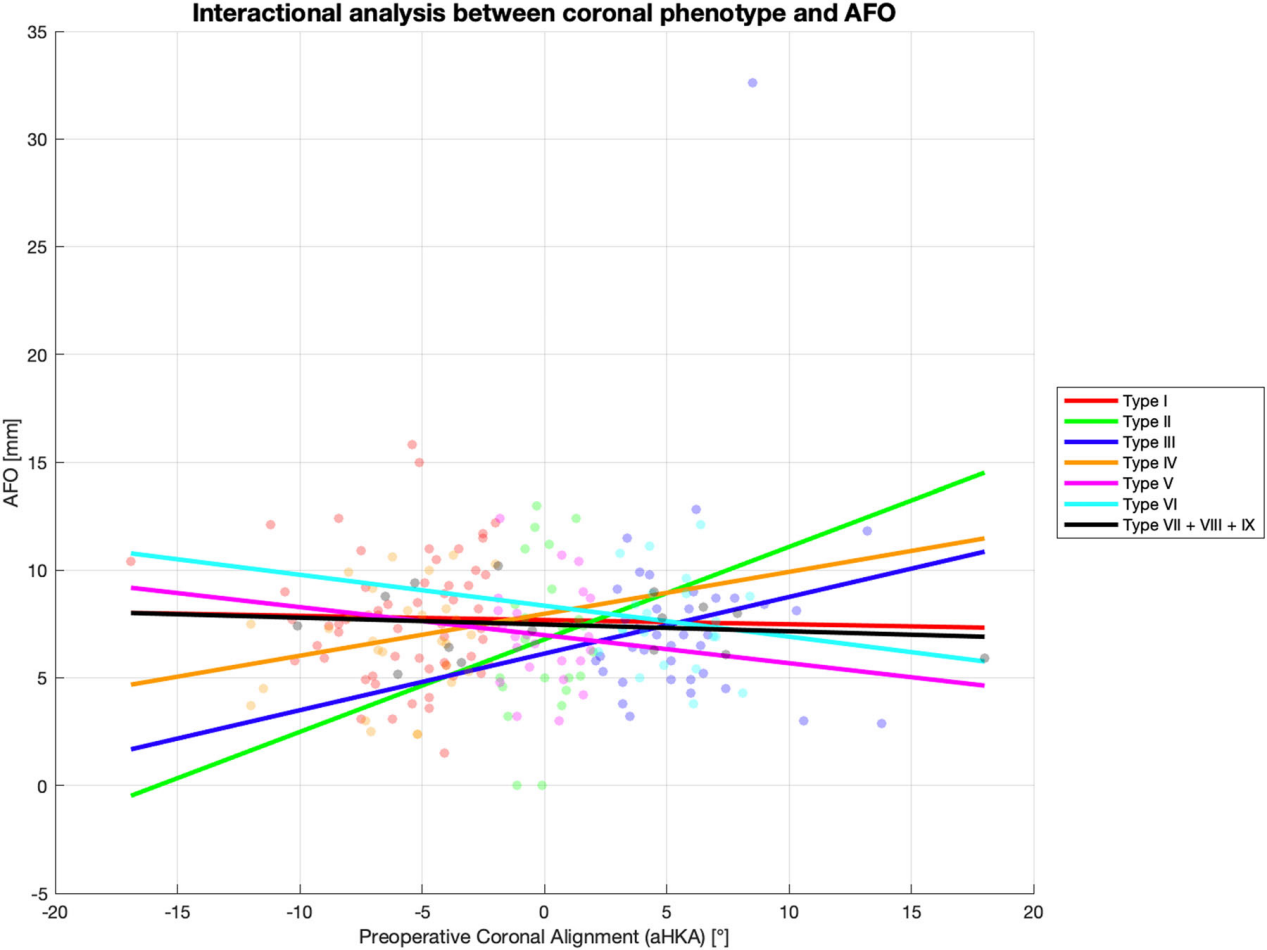
Interaction analysis between sagittal femoral morphology and CPAK classification. Scatter plots showing the relationship between coronal alignment (aHKA) and anterior femoral offset (AFO), stratified by CPAK types. No clear interaction patterns were observed across groups. aHKA, arithmetic hip–knee–ankle angle; CPAK, coronal plane alignment of the knee.

**Figure 2:**
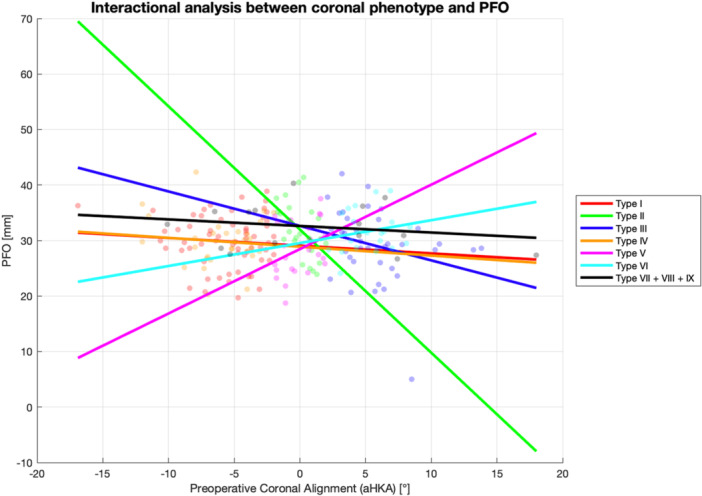
Interaction analysis between sagittal femoral morphology and CPAK classification. Scatter plots showing the relationship between coronal alignment (aHKA) and posterior femoral offset (PFO), stratified by CPAK types. No clear interaction patterns were observed across groups. aHKA, arithmetic hip–knee–ankle angle; CPAK, coronal plane alignment of the knee.

**Figure 3:**
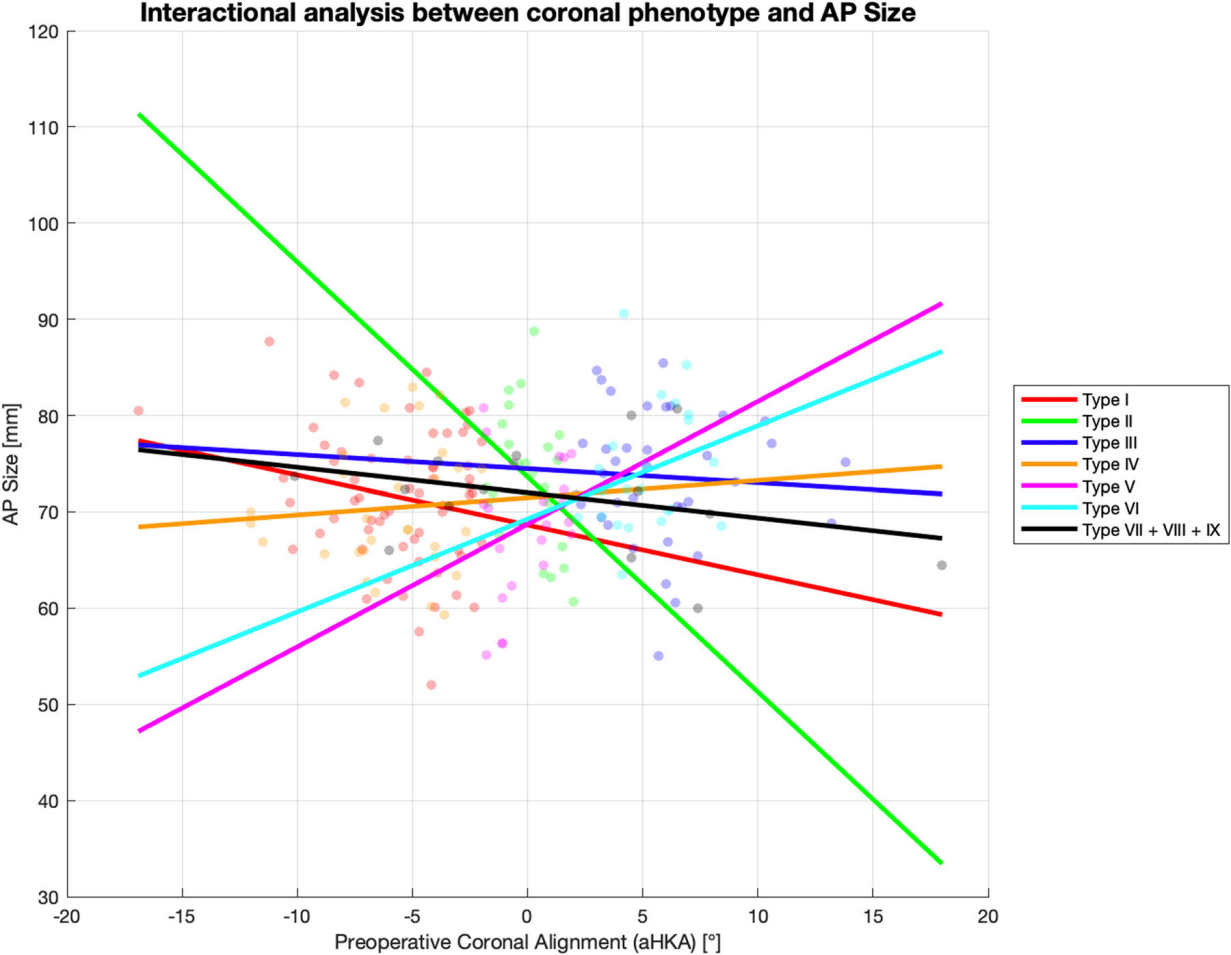
Interaction analysis between sagittal femoral morphology and CPAK classification. Scatter plots showing the relationship between coronal alignment (aHKA) and anteroposterior femoral size (AP), stratified by CPAK types. No clear interaction patterns were observed across groups. aHKA, arithmetic hip–knee–ankle angle; CPAK, coronal plane alignment of the knee.

Detailed results are presented in Table 2 and Figures 1, 2, 3.

**Table 2:** Association between sagittal femoral morphology parameters and coronal alignment classifications.

Coronal parameter	Sagittal parameter	*p*
CPAK	AFO	0.779
	PFO	0.127
	AP	0.124
FKP	AFO	0.622
	PFO	0.409
	AP	0.196
aHKA	AFO	0.910
	PFO	0.881
	AP	0.145
JLO	AFO	0.628
	PFO	0.392
	AP	0.330

Abbreviations: aHKA, arithmetic hip–knee–ankle angle; AFO, anterior femoral offset; AP, anteroposterior femoral size; CPAK, coronal plane alignment of the knee; FKP, femoral knee phenotype; JLO, joint line obliquity; PFO, posterior femoral offset.

### Interaction analysis between sagittal femoral morphology and coronal phenotypes

No significant interaction effects were observed between sagittal femoral morphology and CPAK classification. Interaction terms were not statistically significant for AFO (*p* = 0.874), PFO (*p* = 0.067) and AP size (*p* = 0.363). Although PFO showed a trend toward significance, all models demonstrated low explanatory power (*R* ^2^ ≤ 0.14).

Similarly, no statistically significant interaction effects were identified between sagittal femoral morphology and FKP. Specifically, interaction terms were not significant for AFO (*p* = 0.886), PFO (*p* = 0.886) and AP size (*p* = 0.865), with all models demonstrating negligible explained variance (*R* ^2^ ≈ 0.001–0.002).

**Figure 4:**
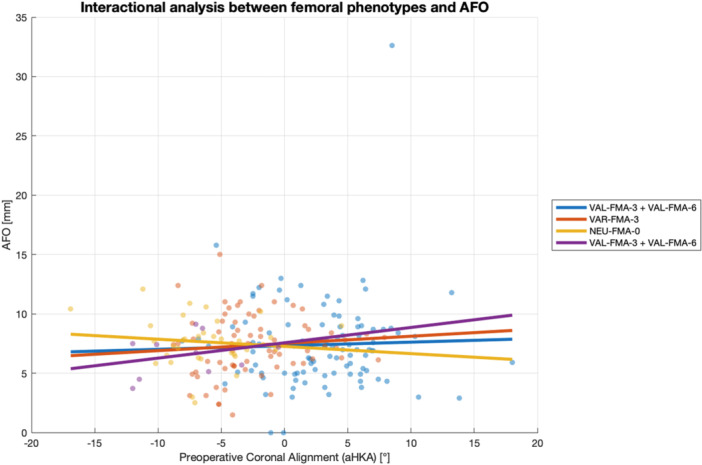
Interaction analysis between sagittal femoral morphology and femoral knee phenotypes (FKPs). Scatter plots showing the relationship between coronal alignment (aHKA) and anterior femoral offset (AFO), across FKP groups. No interaction effect was identified. aHKA, arithmetic hip–knee–ankle angle.

**Figure 5:**
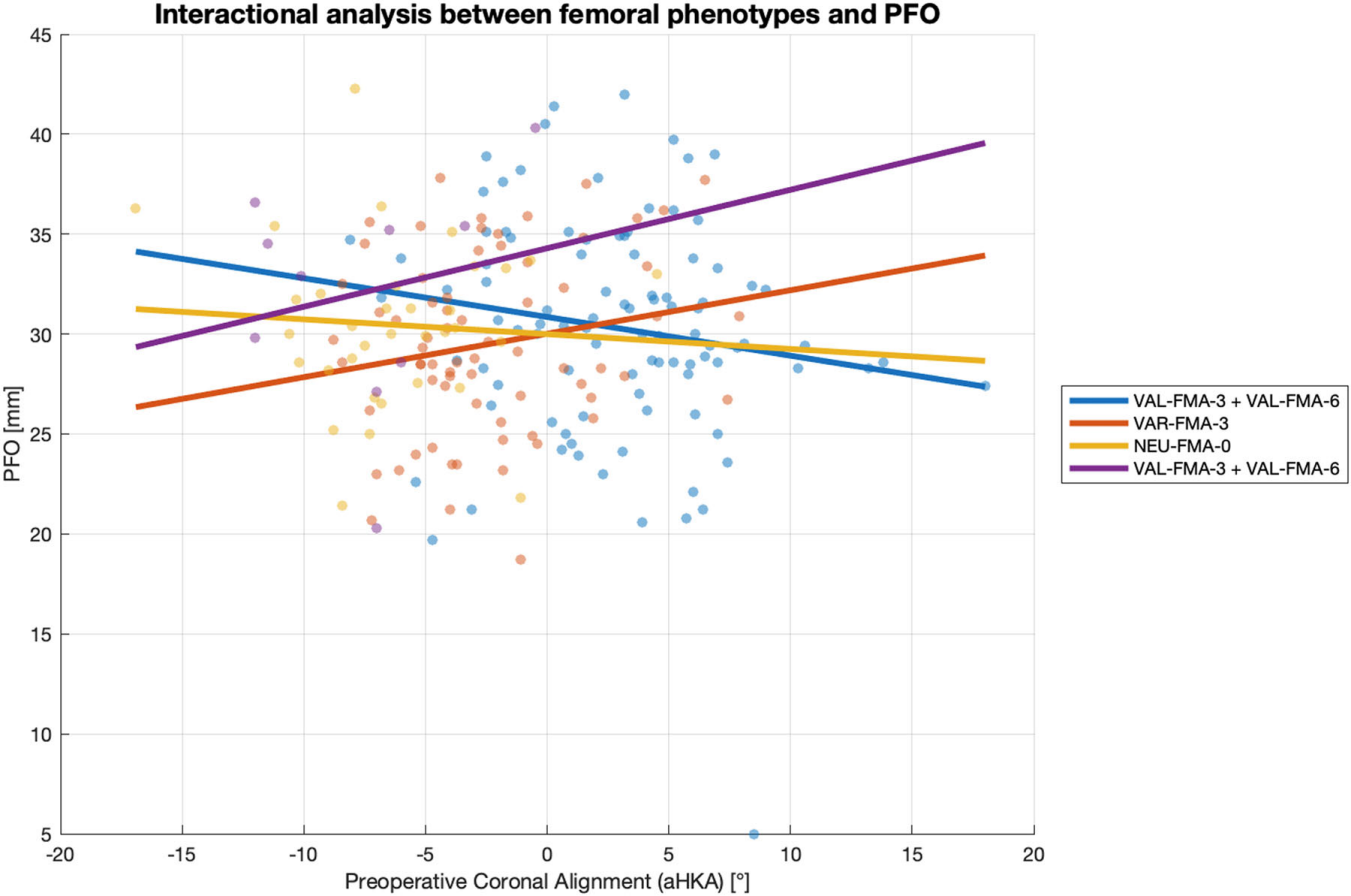
Interaction analysis between sagittal femoral morphology and femoral knee phenotypes (FKPs). Scatter plots showing the relationship between coronal alignment (aHKA) and posterior femoral offset (PFO), across FKP groups. No interaction effect was identified. aHKA, arithmetic hip–knee–ankle angle.

**Figure 6:**
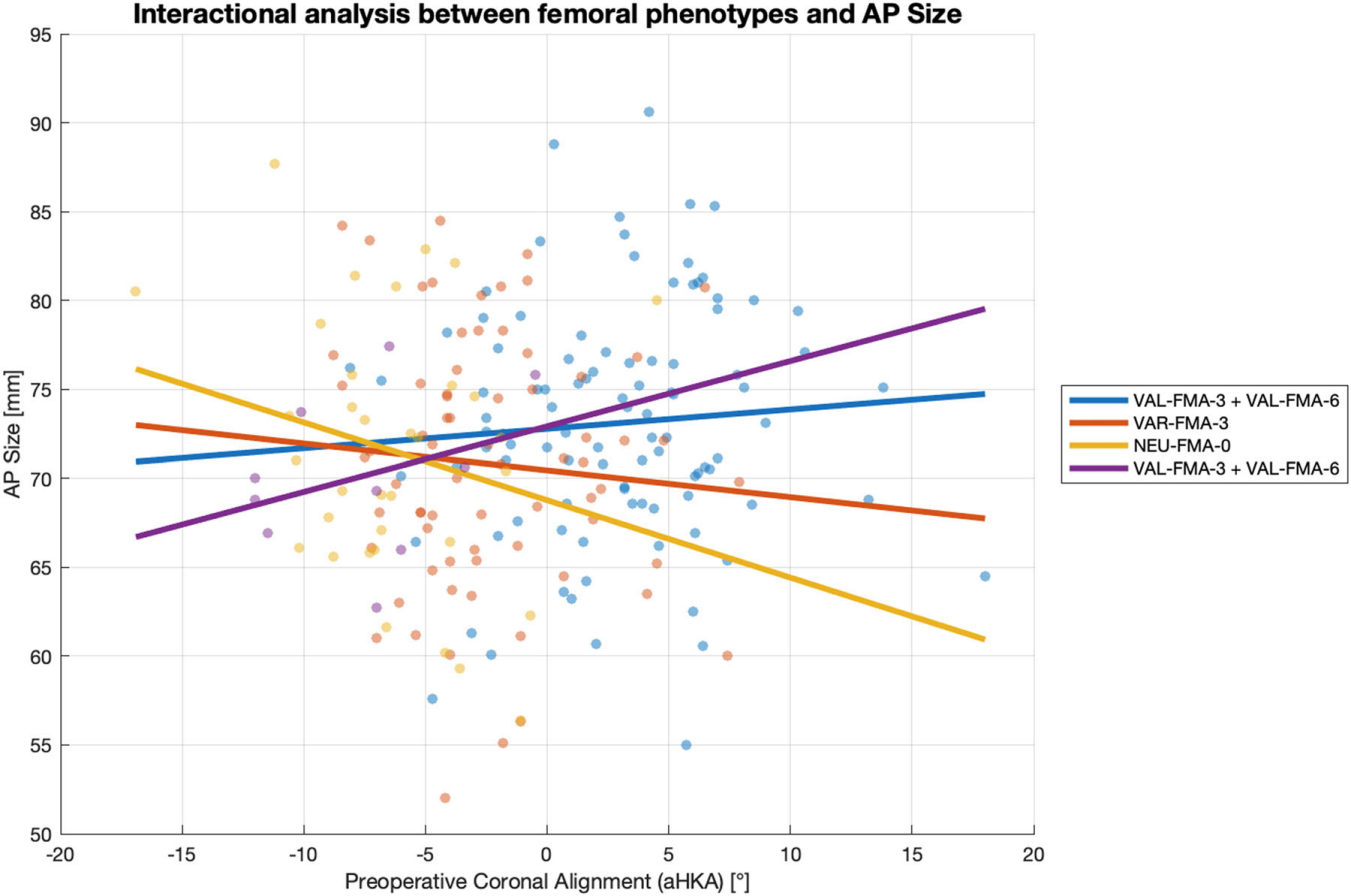
Interaction analysis between sagittal femoral morphology and femoral knee phenotypes (FKPs). Scatter plots showing the relationship between coronal alignment (aHKA) and anteroposterior femoral size (AP), across FKP groups. No interaction effect was identified. aHKA, arithmetic hip–knee–ankle angle.

Detailed results are presented in Table 3 and Figures 4, 5, 6.

**Table 3:** Interaction analysis evaluating whether coronal alignment phenotypes influence the distribution of sagittal femoral morphology parameters.

Coronal classification	Parameter	Interaction *p*	*R* ^2^
CPAK	AFO	0.874	0.040
	PFO	0.067	0.144
	AP	0.363	0.110
FKP	AFO	0.886	0.001
	PFO	0.886	0.001
	AP	0.865	0.002

*Note*: Values reported as interaction *p* values and coefficients of determination (*R* ^2^).

Abbreviations: AFO, anterior femoral offset; AP, anteroposterior femoral size; CPAK, coronal plane alignment of the knee; FKP, femoral knee phenotype; PFO, posterior femoral offset.

## DISCUSSION

The main finding of the present study is that sagittal femoral morphology, as described by AFO, PFO and AP size, was not significantly associated with coronal alignment phenotypes. No relevant differences were observed across CPAK types, FKP groups or coronal radiographic categories based on aHKA and JLO. Moreover, interaction analyses showed that coronal phenotypes did not modify the distribution of sagittal femoral morphology. Taken together, these findings suggest that sagittal femoral morphology and coronal alignment phenotypes represent distinct anatomical domains, each requiring separate consideration during TKA, and that they cannot be fully understood by focusing only on the static coronal plane.

This observation is clinically relevant in the current era of personalised TKA. Over the last decade, increasing dissatisfaction following technically successful TKA has challenged the concept that a single coronal alignment target can be universally applied to all patients [10, 12, 13, 24]. In this context, classifications such as CPAK and FKP have improved the understanding of native coronal variability by incorporating both overall limb alignment and joint line orientation [1, 16, 21, 26, 27].

These systems represent an important conceptual advance over the traditional neutral/varus/valgus framework, as they better reflect constitutional anatomy and may help guide individualised alignment strategies. However, their structure remains inherently coronal. They describe frontal plane morphology but do not account for sagittal anatomy, rotational morphology or the dynamic interaction between bony structures and soft tissues [5, 14, 28].

The present results support this limitation. Although CPAK and FKP effectively describe coronal variability, they were not associated with AFO, PFO or AP size in the current cohort. These findings further support the concept that knee morphology should be evaluated using a multiplanar rather than a purely coronal framework [14, 19, 20].

From a biomechanical perspective, the lack of association observed in this study is plausible. Coronal phenotypes reflect the relationship between the femur and tibia in the frontal plane, primarily through limb alignment and JLO. In contrast, sagittal femoral morphology is more closely related to the local geometry of the distal femur and to the regulation of flexion kinematics, femoral rollback and flexion gap behaviour during knee motion. Parameters such as PFO and AFO have been associated with flexion mechanics, patellofemoral behaviour and restoration of the native femoral contour [7, 11, 18, 22, 23, 30, 31]. These features are not necessarily expected to parallel constitutional coronal alignment, particularly in arthritic knees, where coronal deformity may be influenced by cartilage wear and joint space narrowing [10, 14].

The present findings are also consistent with the growing body of literature emphasising that coronal and sagittal dimensions should be evaluated independently in TKA. Recent studies have increasingly challenged ‘mean value thinking’ and have advocated a multidimensional, patient‐specific approach rather than reliance on simplified alignment categories [19]. Similarly, investigations on sagittal reconstruction in robotic‐assisted TKA have shown that variations in sagittal execution, whether expressed as distal femoral flexion, tibial slope or combined flexion, do not necessarily correspond to predictable differences in outcomes based on coronal patterns [2, 4]. Although these studies focused primarily on post‐operative function rather than morphology, they reinforce the broader concept that sagittal parameters behave as partially independent variables within the overall knee phenotype.

Another important implication of the present findings concerns preoperative phenotyping. There is increasing interest in using coronal classifications such as CPAK to identify patients who may benefit from specific alignment philosophies [17]. From a practical standpoint, sagittal femoral morphology should be directly assessed during preoperative planning rather than inferred from coronal phenotype classifications. This supports a comprehensive multiplanar evaluation of knee anatomy, particularly when advanced planning technologies are available [25, 29, 32].

Finally, the absence of statistically significant interaction effects suggests that the relationship between sagittal and coronal domains remains consistent across the phenotype groups analysed in this cohort. Although PFO showed a borderline trend in the CPAK interaction model, the overall explanatory power was low, indicating that any potential association is likely to be limited. These findings support the concept that sagittal femoral morphology and coronal alignment represent largely independent aspects of knee anatomy.

In addition, AP size, AFO and PFO were analysed as absolute radiographic morphologic parameters rather than normalised values. This approach was chosen because the primary objective of the study was to investigate whether sagittal femoral morphology differed across coronal phenotype classifications, rather than to establish size‐adjusted morphologic reference values. Nevertheless, anthropometric factors such as patient height, body size and sex may influence absolute sagittal measurements and should be considered in future investigations.

Several limitations of the present study should be acknowledged. First, this was a retrospective analysis of a prospectively maintained database, and no formal a priori sample size calculation was performed. Second, the analysis was based on standardised radiographic measurements and therefore does not fully capture the three‐dimensional morphology of the distal femur. Third, although the distribution of valgus knees reflected the epidemiology of arthritic knees undergoing TKA, the relatively small valgus subgroup and the limited number of CPAK types VII–IX may have reduced the ability to identify subgroup‐specific associations. However, excluding these underrepresented categories would have prevented evaluation of the complete CPAK and FKP classification systems as originally described, which was the primary objective of the present study. Fourth, subgroup analyses according to sex and ethnicity were not performed. In addition, multivariable analyses adjusting for anthropometric variables were not performed. Furthermore, sagittal morphologic parameters were analysed as absolute radiographic measurements and were not normalised according to anthropometric variables such as height, body size or femoral length. Therefore, a potential influence of patient morphology on the absolute values of AFO, PFO and AP size cannot be excluded. Finally, although PTS was measured as part of the radiographic assessment, the present study was specifically focused on sagittal femoral morphology rather than tibial morphology, and PTS was therefore not included in the primary analysis.

## CONCLUSIONS

Sagittal femoral morphology, as defined by AFO, PFO and AP size, was not associated with CPAK, FKPs or other coronal alignment parameters.

These findings indicate that sagittal femoral morphology represents an independent anatomical domain that cannot be inferred from coronal phenotype classification alone. A multidimensional, patient‐specific assessment is therefore required when planning TKA.

## AUTHOR CONTRIBUTIONS

Luca Andriollo and Roberta Lazzaro had the idea for the article. Luca Andriollo was responsible for writing the manuscript and qualified as corresponding author. Lorenzo Elia, Giulio Calandra, Marina Marescalchi, Antonio Rapisarda and Roberto Perulli were responsible for data acquisition. Michele Alborghetti was responsible for statistical analysis. Luca Andriollo, Rudy Sangaletti and Francesco Benazzo were responsible for conceptualisation and supervised data acquisition and analysis. Francesco Benazzo and Rudy Sangaletti were responsible for reviewing and critically revising the manuscript. All authors have given final approval of the version to be published.

## FUNDING INFORMATION

The authors have no funding to report.

## CONFLICT OF INTEREST STATEMENT

Francesco Benazzo has a consulting contract with Zimmer Biomet. The other authors declare no conflicts of interest.

## ETHICS STATEMENT

This study was conducted in accordance with the Declaration of Helsinki and approved by the local Institutional Review Board (IRB Approval No. NK5022). Written informed consent was obtained from all participants. All patients provided legitimate informed consent.

## Data Availability

The data that support the findings of this study are available from the corresponding author, [LA], upon reasonable request.
